# Caveolae and the oxidative stress response

**DOI:** 10.1042/BST20230121

**Published:** 2023-05-30

**Authors:** Yeping Wu, Ye-Wheen Lim, Robert G. Parton

**Affiliations:** 1 The University of Queensland, Institute for Molecular Bioscience, 4072 Brisbane, Australia; 2 The University of Queensland, Centre for Microscopy and Microanalysis, 4072 Brisbane, Australia

**Keywords:** caveolae, Cavin1, cell death, lipid peroxidation, NRF2, oxidative stress

## Abstract

Oxidative stress is a feature of many disease conditions. Oxidative stress can activate a number of cellular pathways leading to cell death, including a distinct iron-dependent pathway involving lipid peroxidation, termed ferroptosis, but cells have evolved complex mechanisms to respond to these stresses. Here, we briefly summarise current evidence linking caveolae to the cellular oxidative stress response. We discuss recent studies in cultured cells and in an *in vivo* model suggesting that lipid peroxidation driven by oxidative stress causes disassembly of caveolae to release caveola proteins into the cell where they regulate the master transcriptional redox controller, nuclear factor erythroid 2-related factor 2. These studies suggest that caveolae maintain cellular susceptibility to oxidative stress-induced cell death and suggest a crucial role in cellular homeostasis and the response to wounding.

## Introduction; the cellular response to oxidative stress

Oxidative stress is attributable to an imbalance between the production of reactive oxygen species (ROS) and antioxidant defence. This phenomenon is associated with a wide range of diseases and physiological processes, but its complex effects are particularly well illustrated in cancer. The increased oxidative stress characteristic of highly metabolically active cancer cells can damage DNA and exacerbate inflammation, with a potential role in transformation, tumour cell growth, invasion, and metastasis [1]. However, oxidative stress can also trigger cell death, reducing the chance of transformation and tumorigenesis. Oxidative stress is the major cellular stress occurring upon external stimuli such as radiation [2] and is also a feature of tissue damage in response to wounding under normal physiological conditions [3].

ROS are a group of molecules derived from oxygen, including superoxide, hydrogen peroxide, hydroxyl radical, and singlet oxygen [4]. One of the cellular pathways sensitive to ROS is ferroptosis [5,6], a form of cell death, distinct from apoptosis, in which oxidised polyunsaturated phospholipids accumulate in an iron-dependent manner [7–11]. The pathways regulating ferroptosis are still being unravelled, but a number of features of the pathway are now well established. Suppression of ferroptosis through the clearance of lipid peroxides involves glutathione peroxidase 4 (GPX4), an antioxidant defence enzyme that repairs oxidative damage to lipids [12,13]. Both the availability of iron, as a labile iron pool [6,14] and specific membrane lipids are required for ferroptotic sensitivity. The lipid substrates for ferroptosis are surprisingly specific and a crucial enzyme in this process is long-chain acyl-CoA synthetase 4 (ACSL4) [15]. ACSL4 catalyses the incorporation of polyunsaturated fatty acids (PUFAs) into membrane phospholipids. Particularly important in this pathway are two omega-6 PUFAs, adrenic acid (AdA, 22 : 4) and arachidonic acid (AA, 20 : 4), which must be incorporated into phosphatidylethanolamine (PE) to render cells sensitive to ferroptosis, as shown by elegant inhibition and lipid rescue experiments [16]. The regulation of ACSL4 is therefore crucial for ferroptosis; key regulators include PKCbetaII [17] and Hilpda/HIG2 in clear cell carcinoma cells [18]. The latter is thought to link sources of cellular PUFAs stored in lipid droplets to surface ACSL4-dependent formation of PE-AdA and PE-AA. In contrast with the omega-6 PUFAs that are required for ferroptotic sensitivity, monounsaturated fatty acids, such as oleic acid, can inhibit ferroptosis, promoting the survival of metastasising cancer cell [19,20].

A crucial regulator of the oxidative stress response is nuclear factor erythroid 2-related factor 2 (NRF2), a master transcriptional regulator of cellular redox homeostasis that regulates the expression of a multitude of oxidative stress defence proteins [21]. NRF2 levels are kept low in resting cells through ubiquitylation by KEAP1 which targets NRF2 for degradation [22]. Increased NRF2 activity is associated with oncogenesis, promoting tumour progression, metastasis and resistance to anti-cancer therapies [21,23–25]. In the clinic, high NRF2 expression correlates with poor prognosis by helping cancer cells evade cell death [22]. These effects of NRF2, which inhibit ferroptosis [21,24,25], were assumed to be dependent only on the oxidative stress buffering capacity mediated by the transcriptional targets of NRF2. However, recent work suggests that the ability of NRF2 to regulate the labile iron pool in cells, through regulation of ferritinophagy, is a crucial aspect of its function [14].

## Caveolae and the oxidative stress response

**Figure 1.**
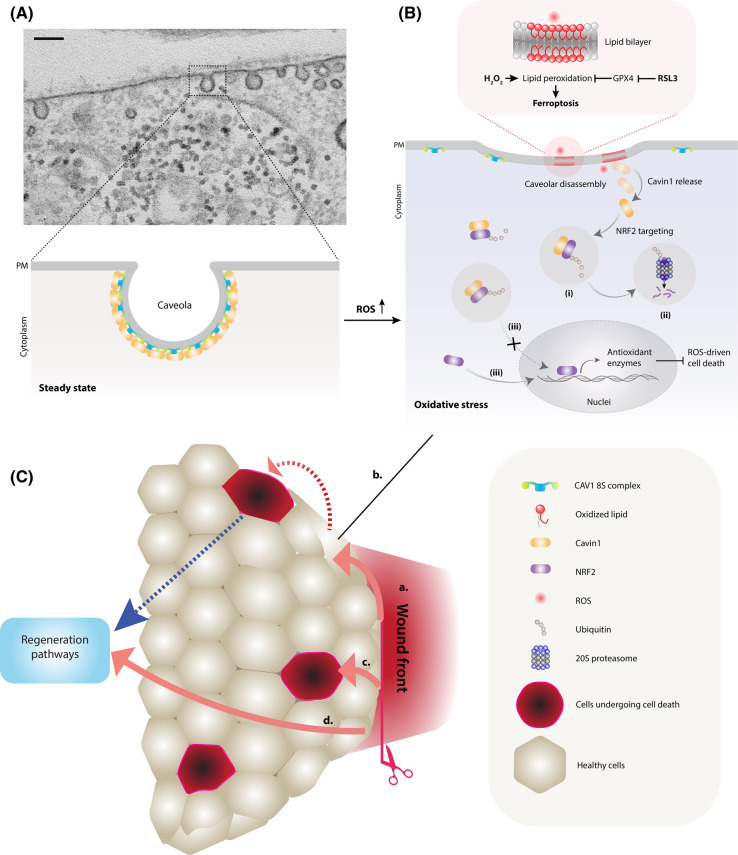
The role of caveolae in cellular response to oxidative stress. (**A**) Top inset: caveolae as viewed by electron microscopy. Scale bar = 100 nm. Bottom inset: An illustration of caveolar structure in a cell under steady state conditions. (**B**) A diagram illustrating caveola-mediated cellular pathway in response to oxidative stress. Exogenous H_2_O_2_ stimulation, or GPX4 inactivation by ferroptosis inducer RSL3 [13,87], promotes ROS accumulation in cells. Excessive ROS-driven lipid peroxidation causes membrane damage and caveolar disassembly, leading to the release of Cavin1. This allows Cavin1 to target NRF2 in the cytosol and promote NRF2 ubiquitination (i), and NRF2 proteasomal degradation (ii). The binding of Cavin1 to NRF2 also inhibits the nuclear import of NRF2 (iii) [78]. The inhibition of NRF2 by Cavin1 through multiple mechanisms reduce the transcriptional levels of NRF2 downstream genes involved in oxidant defence, ultimately promoting ROS-driven cell death including ferroptosis and apoptosis. (**C**) A model for the role of caveolae in cellular oxidative stress response and its bearing in a physiological context of wound healing in the zebrafish. Upon tissue injury (a.), a paracrinal wave of ROS (represented by red arrows) such as H_2_O_2_ signals occur at the local wounding site [78,88]. Cells at steady state near the wound front experience oxidative stress which may trigger ROS-driven cell death (represented by b. and c.; dotted red arrow represents conversion of the state of a cell undergoing oxidative stress to a cell experiencing cell death). Regeneration pathways are subsequently triggered by cell death [79] (blue arrow) or ROS (d.) [3,89,90].

A number of different cellular organelles have been linked to oxidative stress and to ferroptosis, including the peroxisome, the Golgi complex, the endoplasmic reticulum (ER), and mitochondria where ROS are generated (reviewed by Stockwell [7]). Of increasing interest to researchers is the potential role of caveolae, nanoscopic microdomains of the plasma membrane, in the cellular response to oxidative stress. Caveolae are bulb- or flask-shaped invaginations of the plasma membrane that can be readily recognised by electron microscopy (Figure 1A) [26]. The first identified marker of caveolae, caveolin 1 (CAV1), is a small integral membrane protein which forms an oligomeric disc in the cytoplasmic leaflet of the caveolar membrane [27]. Two other caveolin isoforms are expressed in mammalian cells with caveolin 2 (CAV2) coexpressed with, and oligomerising with, CAV1 and caveolin 3 (CAV3) being expressed predominantly in muscle cells [28–30]. CAV1 is not sufficient to form caveolae, but co-operates with peripheral membrane proteins termed cavins [31–36]. Early work on caveolae was focused solely on caveolins and their proposed interactions with many different proteins [37]. In recent years, a shift in focus has occurred with the increased mechanistic understanding that the characterisation of the cavins and other accessory proteins has brought. The current model dictates that caveola formation relies on multiple low-affinity interactions between caveolin proteins, cavin proteins, and membrane lipids to generate a metastable domain [38,39]. This domain can be disassembled in response to various stimuli including an increase in membrane tension, or treatment with UV light, which releases cavins from caveolae into the cytoplasm where they can interact with cellular targets [40–44].

Although a review of the extensive literature linking caveolae to oxidative stress is beyond the scope of this minireview, we will provide a brief overview of relevant and emerging studies. It has been known for some time that caveolae can be modified by oxidative stress, for example by phosphorylation of CAV1 on a key tyrosine, Y14 [45–48]. The tyrosine kinase c-Src, which could be oxidised and activated by ROS [46,49,50], has been shown to be responsible for CAV1 phosphorylation under oxidative stress [45,46,51]. CAV1 phosphorylation on Y14 could be abrogated by incubation with a Src family inhibitor [52]. CAV1 was also shown to be subjected to proteasomal degradation in response to oxidative stress [53,54] with resulting impairment of caveola-dependent cell processes, although caveolae were still detectable by electron microscopy [53]. A functional role for caveolae in the cellular oxidative stress response has also been proposed [55], although a unifying mechanism for how the entire caveolar system (caveolins, cavins, other accessory proteins and membrane lipids) contributes to the oxidative stress response both in cells and whole tissues has been lacking. Earlier studies mainly focussed on CAV1 and many built upon the earlier hypothesis that CAV1 can directly interact with target proteins, a model that is increasingly being questioned [27,48,56,57]. The reported roles of CAV1 are complex as CAV1 expression has been linked to both an increase in oxidative stress or, conversely, to an increase in oxidative buffering capacity. This can be attributed to distinct contexts such as cells with different levels of basal ROS content (e.g. normal cells versus cancer cells [1]), and different concentrations and duration of pro-oxidant treatments in these studies. Alterations in these factors may cause ROS accumulation at different levels and lead to the activation of distinct signalling pathways and biological processes [58,59], thereby affecting the modification and response of CAV1 to oxidative stress. In some studies, CAV1 exhibits an inhibitory effect on oxidative stress. For example, up-regulation of CAV1 was suggested to protect lung cancer cell against excessive ROS-induced cell death, allowing ROS-driven cancer progression [60]. Down-regulation of stromal CAV1 was linked to mitochondrial enzymatic dysfunction and increased ROS production, leading to more aggressive phenotypes of breast cancer cells [61]. CAV1 was also shown to be a negative regulator of ROS production in endothelial cells [62] and in podocytes [63], reducing podocyte cell death [63]. In other studies, however, CAV1 was shown to promote oxidative stress through inhibiting NRF2 (i.e. CAV1 loss increased antioxidant defence) [64,65]. These studies reported an association of a significant pool of NRF2 with CAV1 via the CAV1 scaffolding domain and a caveolin-binding motif in NRF2 [64,65] although the cellular sites of interaction were suggested to be either at the plasma membrane [64] or the nucleus and cytosol [65]. The NRF2-caveolin interaction was lost after 48 h of oxidative stress treatment [64].

Cavin1 has been less extensively studied. Cavin1 deficiency was shown to impair ROS production induced by organ damage in mice [66], indicating a potential role for Cavin1 in regulating the redox state of cells in cancer. Oxidative stress can also activate senescence, where excessive ROS-induced DNA damage response (DDR) signals [67], such as p53, p16 and pRB [68] [69], may lead to a stable cell growth arrest, suppressing tumour development [70]. In this context, Cavin1 has been shown to be activators of the p53/p21 pathway, which places it as a regulator of oxidative stress-induced senescence [71,72].

Another link between caveolae and oxidative stress in cancer is their common interplay with an inflammatory response, a tumorigenesis driver. Inflammatory processes such as excessive ROS production, can be responsible for tumorigenesis through the induction of DNA mutations [73]. In the tumour environment, loss of mesenchymal stromal CAV1 could lead to oxidative stress and drive inflammation [61]. In addition to the direct effect on ROS production, CAV1 has been proposed as a negative regulator of prostaglandin-endoperoxide synthase (PTGS) 2 (also known as COX2) [74], which can be up-regulated by ROS and mediates inflammation [75]. Interestingly, differential regulation of cavin proteins by tumour necrosis factor (TNF), a PTGS2 and inflammation inducer [76], has been observed in mesenchymal stromal cells [77]. This study also revealed a role for Cavin2 as a suppressor of TNF signalling. However, the significance of up-regulated Cavin1 and Cavin3 in TNF-induced inflammation in cancer is yet to be defined. Taken together, these studies indicate that caveolae can act as key redox regulators modulating oxidative stress via multiple processes that are involved in cancer progression.

## An integrated model for a role of caveolae in oxidative stress signalling

The discovery that disassembly of caveolae causes cavin proteins to be released into the cytoplasm [41] where they can interact with, and stabilise, target proteins [40] raised the possibility of examining the entire cellular protein complement linked to cavins to discover what pathways are regulated by caveola disassembly. This unbiased approach was made feasible by development of whole cell quantitative proteomic methods which could be applied to genome-edited cells lacking specific cavin proteins [78]. Unexpectedly, this unbiased approach led to new links between caveolae and oxidative stress.

A striking feature of HeLa cells lacking Cavin1 revealed by whole cell quantitative proteomics was the increase in protein levels of over 40 targets of NRF2 [78]. This corresponded to an increased oxidative stress buffering capacity of the Cavin1-null cells and importantly this was also the case for an *in vivo* model of Cavin1 deficiency; zebrafish lacking the two Cavin1 paralogs, Cavin1a and Cavin1b, similarly showed increased resistance to ROS accumulation after treatment with hydrogen peroxide [78]. In both systems, the increased oxidative stress buffering capacity could be attenuated by expression of Cavin1 but not by expression of CAV1, pointing to a primary role of Cavin1 (and presumably caveolae) in the oxidative stress response [78]. Note that this does not rule out a contributory role of CAV1 in the oxidative stress response.

Treatment with hydrogen peroxide represents a convenient but artificial test of the cellular ROS buffering capacity. However, ROS generation is a feature of tissue wounding *in vivo*. ROS generation triggers cell death and this process is required for efficient regeneration, a pathway dubbed the ‘Phoenix-Rising Pathway’ (see Figure 1C) [79]. The development of the zebrafish line lacking Cavin1 paralogs allowed the role of Cavin1 to be tested in an *in vivo* wounding assay [78]. This revealed that loss of Cavin1 caused a reduction in ROS accumulation at the wound site, reduced apoptosis, and significantly impaired epimorphic regeneration. Thus, the lack of the caveolar system impairs the endogenous ROS-induced apoptosis pathway required for efficient tissue regeneration *in vivo*. Rescue studies in the zebrafish showed that this process was dependent on Cavin-1 but not CAV1.

These experiments reveal an evolutionarily conserved role for caveolae in reacting to oxidative stress. However, it raised the question of the function of caveolae in wild-type cells subjected to oxidative stress. Specifically, why does a loss of Cavin1 (and caveolae) cause increased expression of NRF2 target proteins? And how does the caveolar system and Cavin1 specifically respond to oxidative stress? The results strongly suggested a link between Cavin1 and NRF2. In fact, oxidative stress was shown to cause partial caveola disassembly (as indicated by a reduction in caveolae by electron microscopy), release of Cavin1 into the cytosol, and interaction, direct or indirect, with NRF2 as shown by proximity ligation assays and by immunoprecipitation. This interaction was proposed to inhibit NRF2 activation through two mechanisms; firstly, it was shown that in this system Cavin1 was required for efficient ubiquitylation and degradation of NRF2; second, the Cavin1–NRF2 interaction in the cytosol inhibited nuclear import of NRF2 [78] (Figure 1B). It is also important to note the kinetics of this process. Rather than long-term treatment with oxidising agents over hours and days, the effect of oxidative stress in these studies occurred on the timescale of a few minutes showing a very rapid response to these stimuli. The combined effect of Cavin1 loss was therefore to rapidly increase NRF2 levels and activity.

In this model (Figure 1), Cavin1 released from caveolae in response to oxidative stress can prevent NRF2 activating its targets and so acts as a ‘suicide switch’ to cause cell death and remove the potentially DNA-damaged cells from the population, as observed in the zebrafish wounding assay. Cells lacking Cavin1 showed significantly increased resistance to ferroptosis triggered by inhibition of GPX4 [78] suggesting that caveolae may represent another branch of the ferroptotic regulatory network.

A final question relates to the signal that causes caveola disassembly and the release of cavins for interaction with NRF2. Specific induction of lipid peroxidation was shown to be sufficient to cause cavin release and association with NRF2 [78]. Consistent with this, inhibition of lipid peroxidation blocked cavin release and association of Cavin1 with NRF2. This raises the intriguing possibility that caveolae have evolved to respond to oxidative stress owing to their specialised lipid composition required for their formation and serving as sites for lipid peroxidation. This interesting model is speculative at present but can now be tested.

## Conclusions and future directions

These studies delineate a novel pathway from cell surface caveolae to NRF2 and ferroptosis which involves lipid peroxidation. However, it opens up a number of new questions and avenues for investigation. Recent studies revealed that cell swelling caused by increased membrane permeability and sodium influx is an essential step leading to ferroptotic cell rupture [80,81]. It was also found that this cell swelling process increased tension on the plasma membrane [80]. Previous work showed that membrane stretch by osmotic swelling drives caveolar disassembly and the release of cavins [41,42,44]. This raises a possibility that in addition to lipid peroxidation, increased membrane tension upon cell swelling may also contribute to caveola disassembly and Cavin1 release during ferroptosis induction. There are clearly many different stimuli that cause caveola disassembly and yet it seems unlikely that all would lead to cavin-mediated inhibition of NRF2. One possibility is that a network of post-translational modifications may fine-tune cavin target recognition. It is also unclear how lipid peroxidation leads to disassembly of caveolae. Specific lipids required for caveola formation may be sites for peroxidation causing destabilisation of caveolae. Finally, there are considerable implications for understanding disease conditions associated with loss or dysfunction of caveolae. NRF2 has been extensively studied as a therapeutic target in oxidative stress-involved neurodegenerative diseases [82] and in radio-/chemo-resistant cancer [83,84]. There have been significant advances in drug development based on the mechanisms of NRF2 activation and inhibition [85,86]. The identification of Cavin1 as a binding protein and regulator of NRF2 expands the knowledge of NRF2 signalling and could potentially contribute to therapeutic strategies aimed at the pharmacologic regulation of NRF2 and redox status in cells.

## Perspectives

- Oxidative stress is ubiquitous in biological systems and is a key element in various physiopathological processes. Disruption of caveolae has been linked to an aberrant oxidative stress response, a contributing factor in disease progression.
- Caveolae, composed of specific lipids and accessory proteins, are crucial in sensing and regulating oxidative stress. In the sensing stage, peroxidation of membrane lipids triggers the disassembly of caveolae to release the accessory protein Cavin1 into the cytosol. Redistributed Cavin1 in turn exerts a regulatory role in oxidative stress by inhibiting NRF2. This cellular pathway is essential for mediating oxidative stress-driven cell death including apoptosis and ferroptosis.
- Future investigations into caveola signalling under oxidative stress should focus on (1) dissecting the mechanisms of caveola disassembly upon lipid peroxidation; (2) identifying post-translational modifications required for Cavin1 release and target recognition; (3) identifying the binding sites involved in the Cavin1–NRF2 interaction; (4) exploring the physiological relevance of the CAVIN1–NRF2–ROS axis in other oxidative stress-mediated biological processes.

## Open Access

Open access for this article was enabled by the participation of the University of Queensland in an all-inclusive *Read & Publish* agreement with Portland Press and the Biochemical Society under a transformative agreement with CAUL.

## Abbreviations

AA: arachidonic acid
ACSL4: acyl-CoA synthetase 4
CAV1: caveolin 1
GPX4: glutathione peroxidase 4
NRF2: nuclear factor erythroid 2-related factor 2
PE: phosphatidylethanolamine
PUFAs: polyunsaturated fatty acids
ROS: reactive oxygen species
TNF: tumour necrosis factor
